# Development of a minimally invasive simultaneous estimation method for quantifying translocator protein binding with [^18^F]FEPPA positron emission tomography

**DOI:** 10.1186/s13550-023-00950-1

**Published:** 2023-01-12

**Authors:** Praveen Dassanayake, Udunna C. Anazodo, Linshan Liu, Lucas Narciso, Maryssa Iacobelli, Justin Hicks, Pablo Rusjan, Elizabeth Finger, Keith St Lawrence

**Affiliations:** 1 Department of Medical Biophysics, University of Western Ontario, London, ON Canada; 2 Lawson Health Research Institute, 268 Grosvenor St, London, ON N6A 4V2 Canada; 3 Department of Neurology and Neurosurgery, McGill University, Montréal, QC Canada; 4 Department of Clinical Neurological Sciences, University of Western Ontario, London, ON Canada; 5 Douglas Research Centre, Human Neuroscience Division, Montréal, QC Canada; 6 Department of Psychiatry, McGill University, Montréal, QC Canada

**Keywords:** Positron emission tomography, PET/MR, Translocator protein (TSPO) imaging, Simultaneous estimation method, Kinetic modeling, Image-derived input function

## Abstract

**Background:**

The purpose of this study was to assess the feasibility of using a minimally invasive simultaneous estimation method (SIME) to quantify the binding of the 18-kDa translocator protein tracer [^18^F]FEPPA. Arterial sampling was avoided by extracting an image-derived input function (IDIF) that was metabolite-corrected using venous blood samples. The possibility of reducing scan duration to 90 min from the recommended 2–3 h was investigated by assuming a uniform non-displaceable distribution volume (*V*_ND_) to simplify the SIME fitting.

**Results:**

SIME was applied to retrospective data from healthy volunteers and was comprised of both high-affinity binders (HABs) and mixed-affinity binders (MABs). Estimates of global *V*_ND_ and regional total distribution volume (*V*_T_) from SIME were not significantly different from values obtained using a two-tissue compartment model (2CTM). Regional *V*_T_ estimates were greater for HABs compared to MABs for both the 2TCM and SIME, while the SIME estimates had lower inter-subject variability (41 ± 17% reduction). Binding potential (BP_ND_) values calculated from regional *V*_T_ and brain-wide *V*_ND_ estimates were also greater for HABs, and reducing the scan time from 120 to 90 min had no significant effect on BP_ND_. The feasibility of using venous metabolite correction was evaluated in a large animal model involving a simultaneous collection of arterial and venous samples. Strong linear correlations were found between venous and arterial measurements of the blood-to-plasma ratio and the remaining [^18^F]FEPPA fraction. Lastly, estimates of BP_ND_ and the specific distribution volume (i.e., *V*_S_ = *V*_T_ − *V*_ND_) from a separate group of healthy volunteers (90 min scan time, venous-scaled IDIFs) agreed with estimates from the retrospective data for both genotypes.

**Conclusions:**

The results of this study demonstrate that accurate estimates of regional *V*_T_, BP_ND_ and *V*_S_ can be obtained by applying SIME to [^18^F]FEPPA data. Furthermore, the application of SIME enabled the scan time to be reduced to 90 min, and the approach worked well with IDIFs that were scaled and metabolite-corrected using venous blood samples.

## Background

Activated microglia, the resident immune cells in the brain, can be imaged by positron emission tomography (PET) by targeting mitochondrial 18-KDa translocator protein (TSPO) expression [1–3]. [^11^C]-PK11195 has been the most extensively used TSPO tracer; however, its low specific to non-specific binding ratio has led to the development of second-generation TSPO tracers [4]. One such tracer is [^18^F]FEPPA, which has been used to image neuroinflammation in a number of neurological diseases including Alzheimer’s disease [1]. As with other TSPO tracers, [^18^F]FEPPA quantification can be challenging due to the lack of a suitable reference region since microglia activation can occur throughout the brain. Consequently, the accepted method for quantifying [^18^F]FEPPA uptake is to apply a two-tissue compartment model (2TCM), which requires generating a metabolite-corrected arterial input function (AIF) [2]. In addition to invasive arterial blood sampling, 2–3 h of PET imaging is recommended to estimate the total distribution volume (*V*_T_) with acceptable precession [2].

The simultaneous estimation method (SIME) is an alternative approach that has the advantage of not requiring a reference region to quantify brain uptake. The principle of SIME is to analyze multiple tissue activity curves (TACs) simultaneously to estimate model parameters common to all regions. Initially developed as a modeling approach for estimating the AIF [5], it was subsequently proposed for estimating a common non-displaceable distribution volume (*V*_ND_) across brain regions [6, 7]. Assuming a common *V*_ND_ has the advantage of reducing the number of independent parameters defining the TAC in each brain region. Furthermore, the estimated binding potential relative to a non-displaceable compartment (BP_ND_) is not susceptible to scaling errors in the AIF since it is calculated from the ratio of distribution volumes, namely ${\text{BP}}_{{{\text{ND}}}} = \left( {V_{{\text{T}}} - V_{{{\text{ND}}}} } \right)/V_{{{\text{ND}}}}$ [6, 8]. This advantage is well suited to applications involving either population-based or image-derived input functions, which are prone to scaling errors. Indeed, an initial application to TACs and metabolite-corrected AIFs for [^11^C]PBR28, a second-generation TSPO tracer, showed the ability of SIME to distinguish between high- and mixed-affinity binders (HABs and MABs, respectively) [7]. In addition, if the input function is measured, the specific distribution volume (*V*_S_) can be calculated from regional *V*_T_ and global *V*_ND_ estimates: *V*_S_ = *V*_T_ − *V*_ND_. In certain patient populations, differences in *V*_ND_ compared to controls were found, indicating that *V*_S_ may be a more sensitive indicator of TSPO activity than *V*_T_ [8, 9].

Given the potential advantages of SIME, the overall objective of this study was to develop a minimally invasive SIME approach for [^18^F]FEPPA PET imaging. As the first aim (“Accuracy of SIME” section), retrospective data from healthy individuals were used to compare *V*_ND_ and *V*_T_ estimates derived from the 2TCM and SIME to assess the accuracy of the latter [2]. This analysis was conducted separately for HABs and MABs given the expected differences in TSPO binding. The second aim was to investigate if the scanning duration could be reduced from the recommended 2–3 h [2] to 90 min without compromising the precision of BP_ND_ estimates obtained with SIME (“Error Analysis” section). Next, experiments were conducted in a porcine model, in which arterial and venous blood samples could be drawn concurrently, to investigate if arterial sampling could be avoided by using venous samples for metabolite correction (“Venous vs. Arterial Metabolite Correction” section). Finally, a feasibility study was conducted, by applying the minimally invasive SIME to data from healthy participants (“Feasibility Study” section). The acquisition time was 90 min and image-derived input functions (IDIFs) were acquired instead of AIFs [10]. Serial venous blood samples were used to scale each IDIF and for metabolite correction. The accuracy of the method was investigated by comparing group-wise regional BP_ND_ and *V*_s_ estimates to those from the retrospective data.

## Methods

### SIME method

As recommended by Ogden et al. [6], the SIME approach for estimating a common *V*_ND_ was performed using a small set of ROIs that exhibited a range of kinetic behaviors. For this purpose, the following ROIs were selected based on observable differences in their TACs: frontal lobe (FL), temporal lobe (TL), cerebellum (CBL), thalamus (THA), insula (INS), and caudate (CAU). The common *V*_ND_ was estimated by minimizing a cost function consisting of the sum of the squared difference between each regional TAC and the 2TCM. An optimization routine (i.e., the MATLAB routine fminsearchbnd) was used to perform nonlinear fitting for TACs from the six ROIs, each defined by three rate constants (*k*_2_, *k*_3_, *k*_4_), plus a common *V*_ND_. Note that using an optimization routine to minimize the cost function is different from the grid search approach proposed by Ogden et al. [6]; however, the preliminary assessment indicated a mean difference of 2.3 ± 1.6% in *V*_ND_ estimates from the two methods and the former was less time-consuming. With *V*_ND_ defined, the fitting procedure can be applied to any given region to generate best-fit estimates of *k*_2_, *k*_3_, *k*_4_ with $K_{1} = k_{2} V_{{{\text{ND}}}}$. As a demonstration of this procedure, the fitting was repeated in each of the six ROIs individually with *V*_ND_ fixed to the estimated common value.

The 2TCM included a blood volume term that was fixed to 5% of the brain volume [2]. The rate constants were used to determine regional estimates of the total distribution volume: $V_{{\text{T}}} = V_{{{\text{ND}}}} \left( {1 + k_{3} /k_{4} } \right)$, the non-displaceable binding potential: ${\text{BP}}_{{{\text{ND}}}} = \left( {V_{{\text{T}}} - V_{{{\text{ND}}}} } \right)/V_{{{\text{ND}}}}$, and the specific distribution volume: $V_{{\text{S}}} = V_{{\text{T}}} - V_{{{\text{ND}}}}$ [11].

### Accuracy of SIME

The ability to measure *V*_ND_ and regional *V*_T_ accurately with SIME was evaluated using a dataset consisting of 19 healthy volunteers (11 females and 8 males), divided into two groups based on their TSPO polymorphism genotyping: 7 MABs (mean age 48.00 ± 14.33 y) and 12 HABs (mean age 59.17 ± 19.16 y) previously published [12] including 12 subjects from Ref. [2].

#### Data acquisition

Details of the imaging procedures can be found in the previous reports [2, 12]. Briefly, PET data were acquired on a 3D high-resolution research tomograph (HRRT) scanner (CPS/Siemens, Knoxville, TN, USA). The imaging procedure consisted of 180 min (*n* = 12) and 120 min (*n* = 7) of list-mode acquisition following intravenous injection of [^18^F]FEPPA (173 ± 13 MBq). Images were reconstructed into 1 background frame of variable length followed by either 33 or 44 time frames depending on the length of scan (5 × 30, 1 × 45, 2 × 60, 1 × 90, 1 × 120, 1 × 210, and 22 or 33 × 300 s) with a matrix size of 256 × 256 × 207 and voxels of 1.22 × 1.22 × 1.22 mm^3^.

During the PET acquisition, arterial blood was continuously drawn at a rate of 2.5 ml/min for 22.5 min using an automatic blood sampling system and manually sampled at 2.5, 7, 12, 15, 20, 30, 60, 90, 130, and 180 min. The blood samples were used to determine blood-to-plasma ratios (BPRs), which were fitted with a biexponential function. Plasma samples were used to determine relative parent and metabolite concentrations, and a Hill function was used to determine the fraction of unmetabolized [^18^F]FEPPA. These correction functions were applied to the measured blood TAC to generate a metabolite-corrected plasma AIF [1, 2].

For region-of-interest (ROI) analysis, proton-density MR images were acquired on a 1.5 T Sigma scanner (General Electric, Milwaukee, WI). Imaging parameters were slice thickness = 2 mm, repetition time > 5300 ms, echo time = 13 ms, flip angle = 90°, number of excitations = 2, acquisition matrix = 256 × 256, and field of view = 22 cm.

#### Data analysis

The accuracy of SIME was assessed by comparing *V*_ND_ and regional *V*_T_ estimates to those obtained by tracer kinetic modeling. The 2TCM was fit to each regional TAC using a weighted nonlinear least-squares approach based on the MATLAB optimization routine *fmincon* (MATLAB 2018a, MathWorks, Natick, MA). As with the SIME approach, the 2TCM included a fixed blood volume of 5% of brain tissue. The distribution volumes were derived from the estimated rate constants *K*_1_, *k*_2_, *k*_3_, and *k*_4_ as described previously for SIME. Both SIME and the 2TCM were applied to TACs of 120-min duration for the six ROIs (FL, TL, Ceb, Tha, Ins, Cau). The analysis was conducted separately for MABs and HABs. To investigate if reducing the scan duration would affect the accuracy of SIME, the procedure was repeated for a scan duration of 90 min. To evaluate the dependency of SIME on the chosen number of ROIs, the analysis for the 90-min scan time was repeated for all possible combinations of four and five ROIs from the original six regions.

### Venous versus arterial metabolite correction

Animal experiments were conducted for the purpose of comparing venous metabolite correction against concurrent arterial metabolite correction; as such, these experiments did not involve PET scanning. The experiments were conducted according to the guidelines of the Canadian Council on Animal Care and approved by the Animal Use Committee at Western University. Eight female juvenile pigs were obtained from a local supplier (age range 8–10 weeks, mean weight 19.6 ± 3.0 Kg). Under 3% isoflurane anesthesia, the animals were tracheotomized and mechanically ventilated on a mixture of oxygen and medical air. Catheters were inserted into the cephalic veins for [^18^F]FEPPA injections and into the femoral arteries for intermittent blood sampling, measurement of PaCO_2_ and arterial O_2_ tension, monitoring of blood pressure, and measuring the AIF. After surgical preparation, the isoflurane was reduced to between 1 and 3%; a pulse oximeter was used to monitor arterial oxygen saturation and heart rate. At the end of the experiment, the animals were euthanized according to animal care guidelines.

Following a bolus iv injection of [^18^F]FEPPA (5 MBq/kg), venous and arterial blood samples were acquired manually at 2.5, 7, 12, 20, 30, 45, 60, 90, 120, and 180 min post-injection. Each aliquot was centrifuged at 1500×*g* for 5 min to extract the plasma, and the concentrations of radioactivity in whole-blood and plasma samples were measured using a high-purity germanium well counter. The unmetabolized fraction of [^18^F]FEPPA was measured by solid-phase extraction chromatography. The BPRs were generated by fitting a biexponential function, and a Hill function was used to estimate the percentage of unmetabolized [^18^F]FEPPA [2].

## Feasibility study

The study was approved by the Western University Health Sciences Research Ethics Board and conducted in accordance with the Declaration of Helsinki’s ethical standards. Fourteen neurologically healthy participants (7 females and 7 males) were recruited through advertisements and the volunteer pool at the Cognitive Neurology and Aging Brain Clinic at Parkwood Hospital (St Joseph’s Health Care London). Participants were divided into two groups based on their TSPO polymorphism genotyping: 6 MABs (mean age 71.94 ± 12.09 y) and 8 HABs (mean age 75.57 ± 16.88 y).

## Data acquisition

Participants were scanned for 90 min on a fully integrated PET/MRI system (Biograph mMR, Siemens Healthcare GmbH, Erlangen, Germany) using a 16-channel (12 head, 4 neck array) PET-compatible RF coil. Following the intravenous administration of [^18^F]FEPPA (209.20 ± 52 MBq), 90 min of list-mode PET data was acquired. Dynamic images were reconstructed into 52 time frames (1 × 20 s, 12 × 5 s, 8 × 15 s, 4 × 30 s, 5 × 60 s, 10 × 120 s, 11 × 300 s, and 1 × 279 s) with a matrix size of 344 × 344 × 127 and voxel size of 1.043 × 1.043 × 2.032 using the Siemens e7 tools and an iterative reconstruction algorithm without point-spread function modeling (ordered subset expectation maximization (OSEM) with 3 iterations, 21 subsets and 2 mm Gaussian post-smoothing filter). The PET data were corrected for decay, scatter, and dead time, while attenuation correction was performed using the RESOLUTE method applied to ultrashort echo-time MR images [13]. The imaging protocol included the acquisition of *T*1-weighted structural images using a magnetization-prepared rapid gradient-echo (MP-RAGE) sequence (matrix size: 256 × 256 × 240, voxel size: 0.8 × 0.8 × 0.8 mm, echo time: 2.25 ms, repetition time: 2400 ms, and flip angle: 8°). Venous blood samples for metabolite analysis were manually drawn at the antecubital fossa (ACF). Catheters were inserted left and right ACF in both forearms, one for injection, the other for blood draw. Venous blood samples were collected at 2.5, 7, 12, 20, 30, 45, 60, and 90 min.

## IDIF extraction

For PET quantification using SIME, each participant’s IDIF was extracted using a semi-automated software algorithm that extracts a subject-specific mask of the carotid arteries from high-resolution anatomical MR images, which were subsequently registered to corresponding PET images [10]. The whole-blood IDIF extracted from the carotid mask was corrected for partial volume errors and spill-in contamination [14, 15] and scaled to whole-blood venous samples acquired at 45, 60, and 90 min. Venous blood samples collected were analyzed to calculate BPRs and remaining [^18^F]FEPPA fractions according to a previously published method [2]. Following the procedure used in the animal validation experiments, radioactivity in whole-blood and plasma samples was measured using a well counter and the unmetabolized [^18^F]FEPPA fraction was measured by chromatography. A biexponential function was used to determine the BPRs and a Hill function was used to estimate the unmetabolized [^18^F]FEPPA fraction. These measurements were calculated for each subject individually. A metabolite-corrected plasma IDIF was generated by applying the correction functions for BRP and the remaining [^18^F]FEPPA fraction to the whole-blood IDIF [2].

## Image preprocessing

Dynamic PET images were processed using SPM12 (https://www.fil.ion.ucl.ac.uk/spm/). The images were realigned, registered to the anatomical image, skull stripped, and spatially normalized to Montreal Neurological Institute (MNI) space using SPM’s unified segmentation-based normalization approach [16]. The six ROIs used for SIME and the 2TCM were defined based on the AAL atlas [17] and extracted using an in-house MATLAB script.

### Error analysis

Numerical simulations were conducted to examine the accuracy and precision of parameter estimates obtained by applying SIME to 90 min of [^18^F]FEPPA data. The analysis was based on data used to determine the accuracy of SIME. First, the residual between the best fit of the 2TCM and a TAC was calculated at each time frame and normalized by the mean activity. The procedure was performed for all regions (i.e., for FL, TL, Ceb, Tha, Ins, and Cau) and across all 19 participants [8, 18]. The average residual for each region and time frame was calculated across participants. Next, one participant’s dataset was selected, and the initial values of *K*_1_, *k*_2_, *k*_3_, and *k*_4_ for each of the six ROIs were defined by the best fit of the 2TCM. Simulations were performed by adding Gaussian noise to the theoretical TACs. The magnitude of the noise at each time frame was scaled to reflect the average residual calculated for that time frame and region. The SIME procedure was repeated 1000 times to generate histograms of best-fit estimates of *k*_2_, *k*_3_, and *k*_4_ for each region and a common *V*_ND_, which were subsequently used to calculate *V*_T_, BP_ND_, and *V*_S_. The precision of each parameter was defined by the coefficient of variation (COV) of the histogram and the accuracy by the agreement between the mean estimate and the input value.

### Statistics

Analysis of variance (ANOVA) was conducted to compare HABs and MABs *V*_T_ estimates determined by the 2TCM and SIME, with ROI as the within-subject variable and genotype as the between-subject variable. Similarly, an ANOVA was used to determine differences between *V*_ND_ estimates obtained by the two methods. Repeated-measures ANOVAs were used to compare regional BP_ND_ estimates for the two scan durations (90 and 120 min), as well as the corresponding *V*_ND_ estimates. Similarly, repeated-measures ANOVAs were used to assess the effects of reducing the number of ROIs on the *V*_ND_ and regional BP_ND_ estimates. Differences in inter-subject variability were assessed using an *F* test to compare variances. The analysis was performed for *V*_T_ estimates from the 2TCM and SIME, as well as BP_ND_ and *V*_ND_ estimates from SIME for the two scan durations.

Pearson’s correlations analysis was conducted between venous and arterial blood-to-plasma ratios (BPR) and remaining [^18^F]FEPPA fractions. The difference in the regression slope from a value of one was performed using a paired *t* test. For the feasibility study, a two-way repeated-measures ANOVA was used to compare BP_ND_ and *V*_S_ estimates from the group involving IDIFs to the group involving AIFs. Each analysis was conducted over six ROIs with binding affinity as the between-subject variable. All statistical tests involving multiple comparisons included Bonferroni corrections. Analysis was performed in SPSS (IBM, Armonk, NY, USA, version 27) using the *F* test to assess the homogeneity of variance. Statistical significance was assessed based on *p* < 0.05. All values are reported as mean ± standard deviation (SD).

## Results

### Accuracy of SIME

**Fig. 1:**
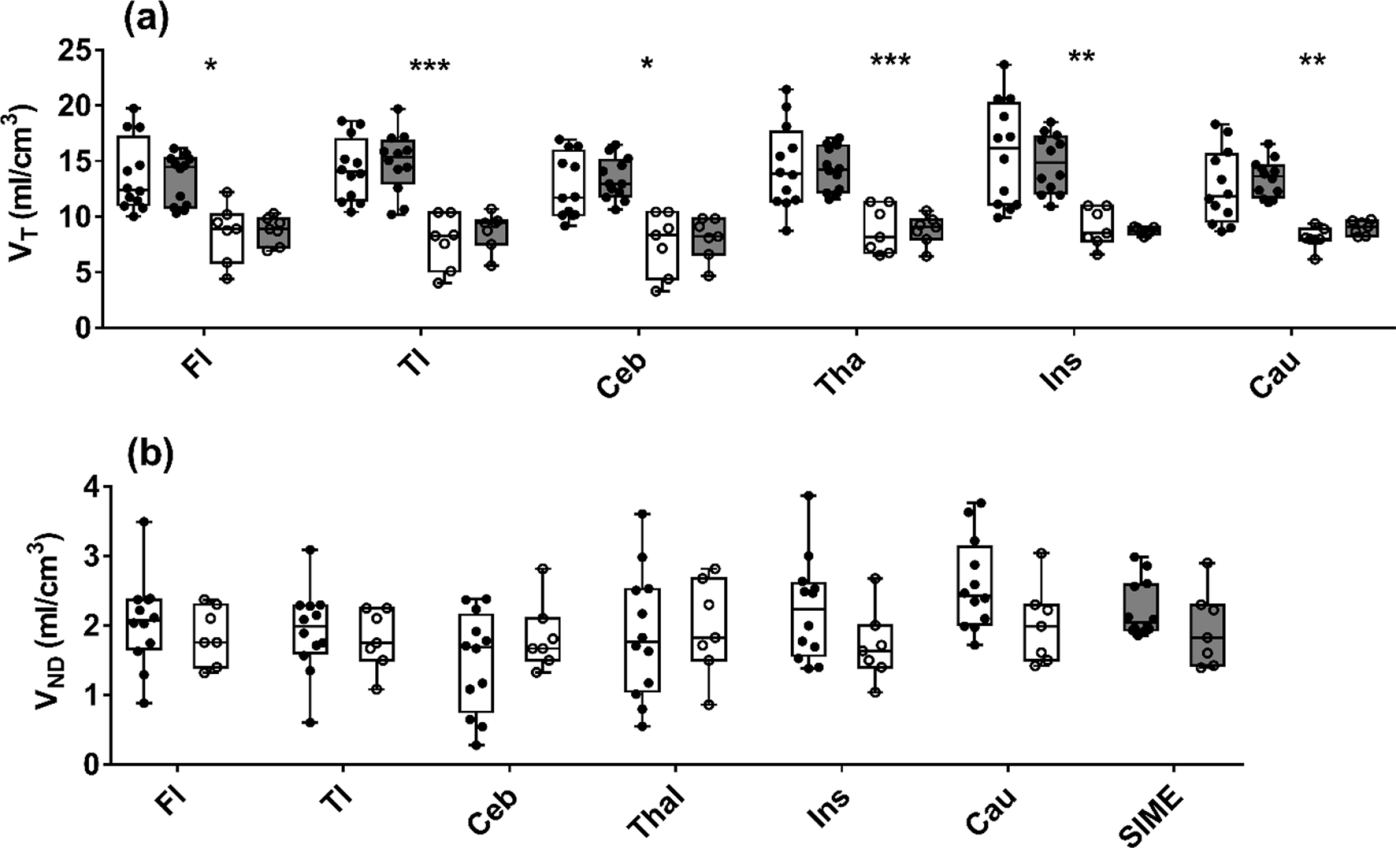
**a** Regional V_T_ estimates from the 2TCM (open box plots) and SIME (gray box plots). **b** Corresponding estimates of regional *V*_ND_ from the 2TCM and common *V*_ND_ from SIME. Results have been separated into HABs (filled circles) and MABs (open circles). Frontal lobe: Fl, Temporal lobe: Tl, Cerebellum: Ceb, Thalamus: Thal, Insula: Ins, and Caudate: Cau. Data are presented as box–whisker plots with the median indicated by the black line. Significant between HABs and MABs are indicated by asterisk (*p < 0.05, **p < 0.01 and ***p < 0.001)

Average estimates of *V*_T_ and *V*_ND_ from the 2TCM and SIME are presented in Fig. 1a, b, respectively. Mean estimates of regional *V*_T_ are provided in Table 1. No statistical differences in *V*_T_ estimates from the two methods were found in any of the ROIs; however, *V*_T_ was significantly higher for HABs compared to MABs for both the 2TCM (30 ± 4%) and SIME (28 ± 8%). No significant difference was found between *V*_ND_ obtained by SIME (2.12 ± 0.47 ml/cm^3^) and the average of the six ROIs from the 2TCM (1.98 ± 0.69 ml/cm^3^). No significant difference in *V*_ND_ was found between HABs and MABs by either method. The inter-subject variability for regional *V*_T_ was significantly lower for SIME compared to the 2TCM (41 ± 17%, *p* < 0.01).

**Table 1:** Average estimates of *V*_T_ and BP_ND_ from 19 healthy volunteers with measured AIFs for 120-min scan duration

Brain region	HABs		MABs	
	*V*_T_ (ml/cm^3^)	BP_ND_	*V*_T_ (ml/cm^3^)	BP_ND_
Frontal	13.4 ± 2.1	5.8 ± 1.4	8.8 ± 1.3	2.9 ± 0.5
	*13.7* ± *3.2*		*8.5* ± *2.6*	
Temporal	14.9 ± 2.6	5.6 ± 1.4	8.7 ± 1.7	2.8 ± 0.6
	*14.3* ± *2.8*		*7.8* ± *2.4*	
Cerebellum	13.4 ± 1.8	5.4 ± 1.1	8.0 ± 1.9	2.4 ± 0.8
	*12.7* ± *2.9*		*7.6* ± *2.8*	
Thalamus	14.3 ± 1.9	5.8 ± 1.2	8.9 ± 1.3	2.7 ± 0.8
	*14.5* ± *3.8*		*8.8* ± *2.1*	
Insula	14.9 ± 2.5	5.7 ± 1.2	8.7 ± 0.34	3.1 ± 0.4
	*15.7* ± *4.6*		*9.0* ± *1.7*	
Caudate	13.1 ± 1.6	5.2 ± 1.3	9.0 ± 0.7	2.9 ± 0.6
	*12.68* ± *3.3*		*8.1* ± *1.2*	

*V*_T_ determined by SIME (top) and the 2TCM (bottom, italic), while BP_ND_ was only derived from SIME

**Fig. 2:**
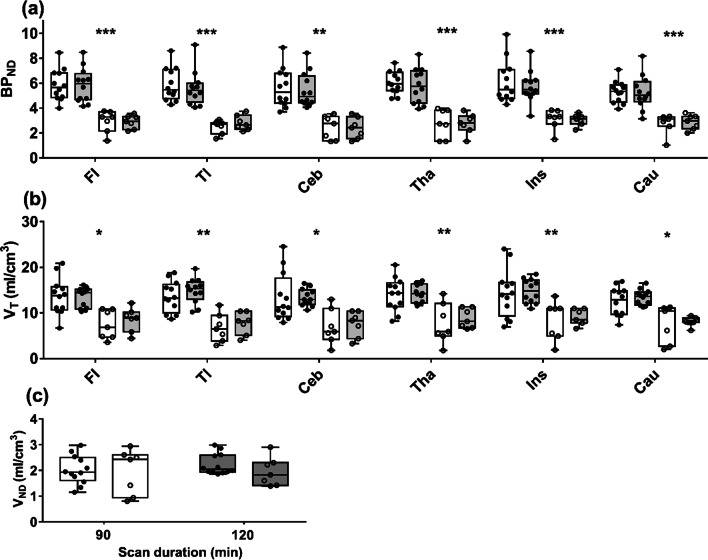
**a** Regional BP_ND_ estimates from SIME for scan time durations of 90 and 120 min (open box plots and dark gray box plots, respectively). **b** Corresponding *V*_T_ estimates and **c** *V*_ND_ estimates for the same scan durations. Data are presented as box–whisker plots with the median indicated by a black line. Results were separated based on binding affinity: filled circles represent HABs and open circles represent MABs. Significant between HABs and MABs are indicated by asterisk (*p < 0.05, **p < 0.01, ***p < 0.001)

Figure 2 presents the estimates of regional BP_ND_, *V*_T_, and brain-wide *V*_ND_ obtained by applying SIME to TACs of 90 and 120 min in duration. The mean values of regional BP_ND_ are provided in Table 1. No significant difference in BP_ND_ for any of the six ROIs was found for the two scan durations. Independent of duration, BP_ND_ was significantly greater for HABs than MABs (average difference of 42 ± 6%). Similarly, no significant changes were observed in the mean *V*_T_ estimates across ROIs and scan durations though *V*_T_ for HABs was significantly greater than that of MABs (average difference of 42 ± 8%). Scan duration had no significant effect on *V*_ND_, and no difference between HABs and MAB was found.

Permutation analysis indicated that reducing the number of ROIs used in SIME to estimate *V*_ND_ (i.e., from 6 to 5 to 4 to 3) did not significantly change the average value: *V*_ND_ = 2.21 ± 0.35, 2.11 ± 0.42 and 2.01 ± 0.48 mL/cm^3^ for 6, 5 and 4 ROIs, respectively. However, SIME failed once the number of ROIs was reduced to 3. Also, reducing the number of ROIs did result in greater inter-subject variability (*F* test, *p* < 0.01). Similar results were found for regional BP_ND_ with a significant increase in inter-subject variability as the number of ROIs decreased from 6 to 5 to 4 (*p* < 0.01).

### Error analysis

**Fig. 3:**
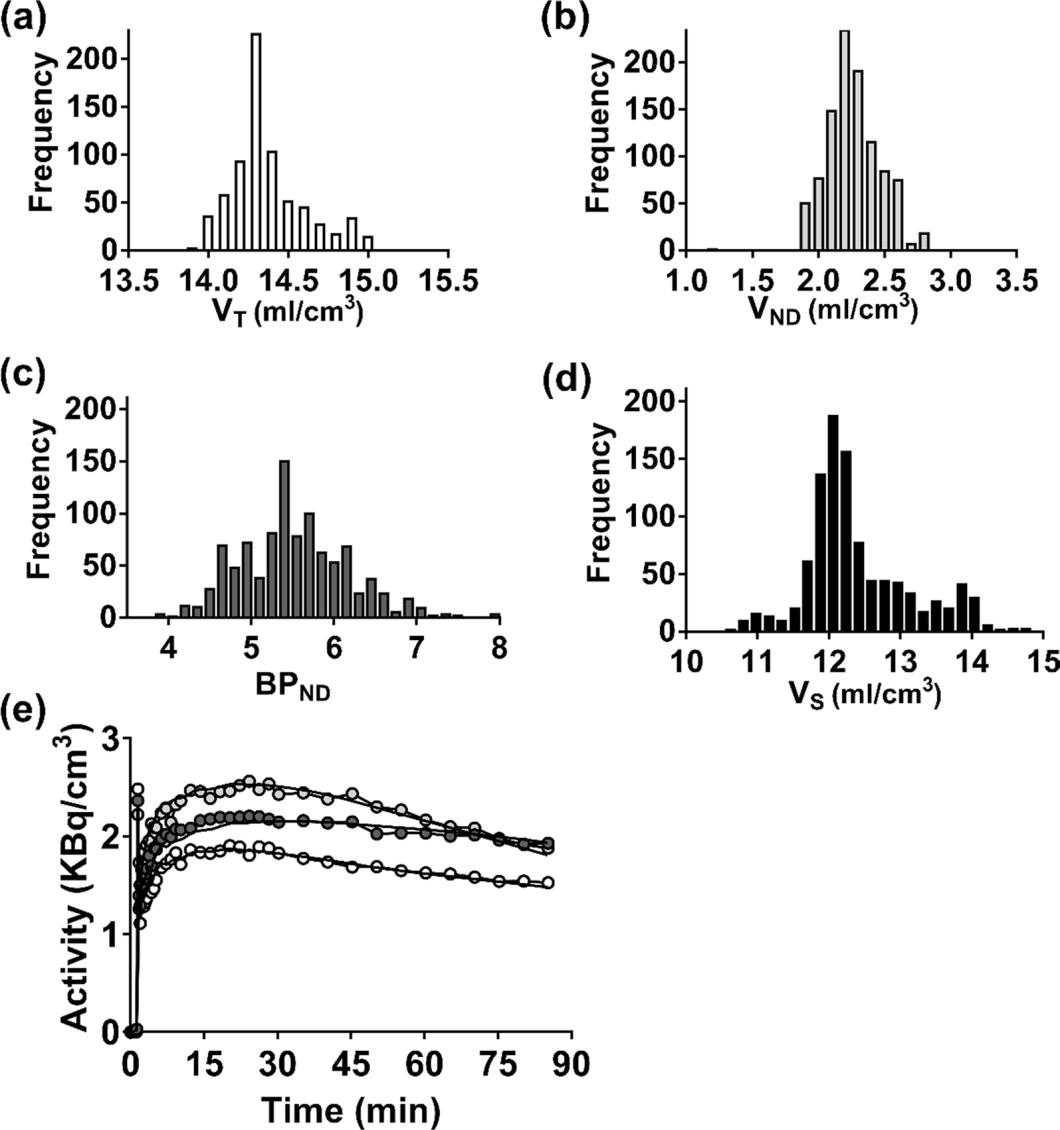
Histograms from 1000 simulations for frontal lobe: **a** *V*_T_ (input = 14.30 and mean = 14.28 (^T^13.4–^S^15.1) ml/cm^3^), **b** *V*_ND_ (input = 2.35 and mean = 2.34 (^T^2.20–^S^2.50) ml/cm^3^), **c** BP_ND_ (input = 5.10 and mean = 5.13 (^T^4.6–^S^5.6)) and **d** *V*_S_ (input = 11.95 and mean = 11.93 (^T^11.1–^S^12.7) ml/cm^3^). **e** Representative simulated TACs for the frontal lobe (dark gray circles), caudate (light gray circles) and cerebellum (open circles), along with the best fit of the two-compartment model from SIME. ^T^25th percentile and ^S^75th percentile

Results of the Monte Carlo simulations for 90-min scan duration are provided in Table 2. Good agreement was found between the input value and the mean of the fitting results for *V*_ND_ (mean difference = 0.4 ± 3%), *V*_T_ (range 0.01 to 1%), *V*_S_ (range 0.07–2%), and BP_ND_ (range 0.7–1%). Corresponding COVs for each parameter were small, ranging from 6 to 10% for BP_ND_ and 7–12% for *V*_S_. Figure 3 shows the histograms of best-fit estimates for the four parameters from the frontal lobe for 90-min scan duration.

**Table 2:** Error analysis results from Monte Carlo simulations for 90-min scan duration

Brain region	True value				Simulated results (Mean ± SD)			
	*V*_ND_ (ml/cm^3^)	*V*_T_ (ml/cm^3^)	*V*_S_ (ml/cm^3^)	BP_ND_	*V*_ND_ (ml/cm^3^)	*V*_T_ (ml/cm^3^)	*V*_S_ (ml/cm^3^)	BP_ND_
Frontal	2.35	14.30	11.95	5.10	2.34	14.28 (^T^13.4–^S^15.1)	11.93 (^T^11.1–^S^12.7)	5.13 (^T^4.6–^S^5.6)
Temporal		15.16	12.81	5.45		15.14 (^T^14.6–^S^15.8)	12.80 (^T^12.3–^S^13.5)	5.51 (^T^5.0–^S^6.0)
Cerebellum		11.69	9.34	3.98		11.52 (^T^10.1–^S^11.8)	9.18 (^T^7.9–^S^9.7)	3.95 (^T^3.3–^S^4.4)
Thalamus		11.80	9.45	4.02		11.78 (^T^11.0–^S^12.1)	9.44 (^T^8.6–^S^9.6)	4.06 (^T^3.5–^S^4.4)
Insula		15.10	12.75	5.42		15.0 (^T^14.3–^S^15.6)	12.66 (^T^12.0–^S^13.1)	5.45 (^T^5.0–^S^5.9)
Caudate		13.80	11.45	4.87		13.80 (^T^12.8–^S^14.8)	11.46 (^T^10.5–^S^12.4)	4.88 (^T^4.7–^S^5.1)

^T^25th percentile and ^S^75th percentile

### Venous versus arterial metabolite correction

**Fig. 4:**
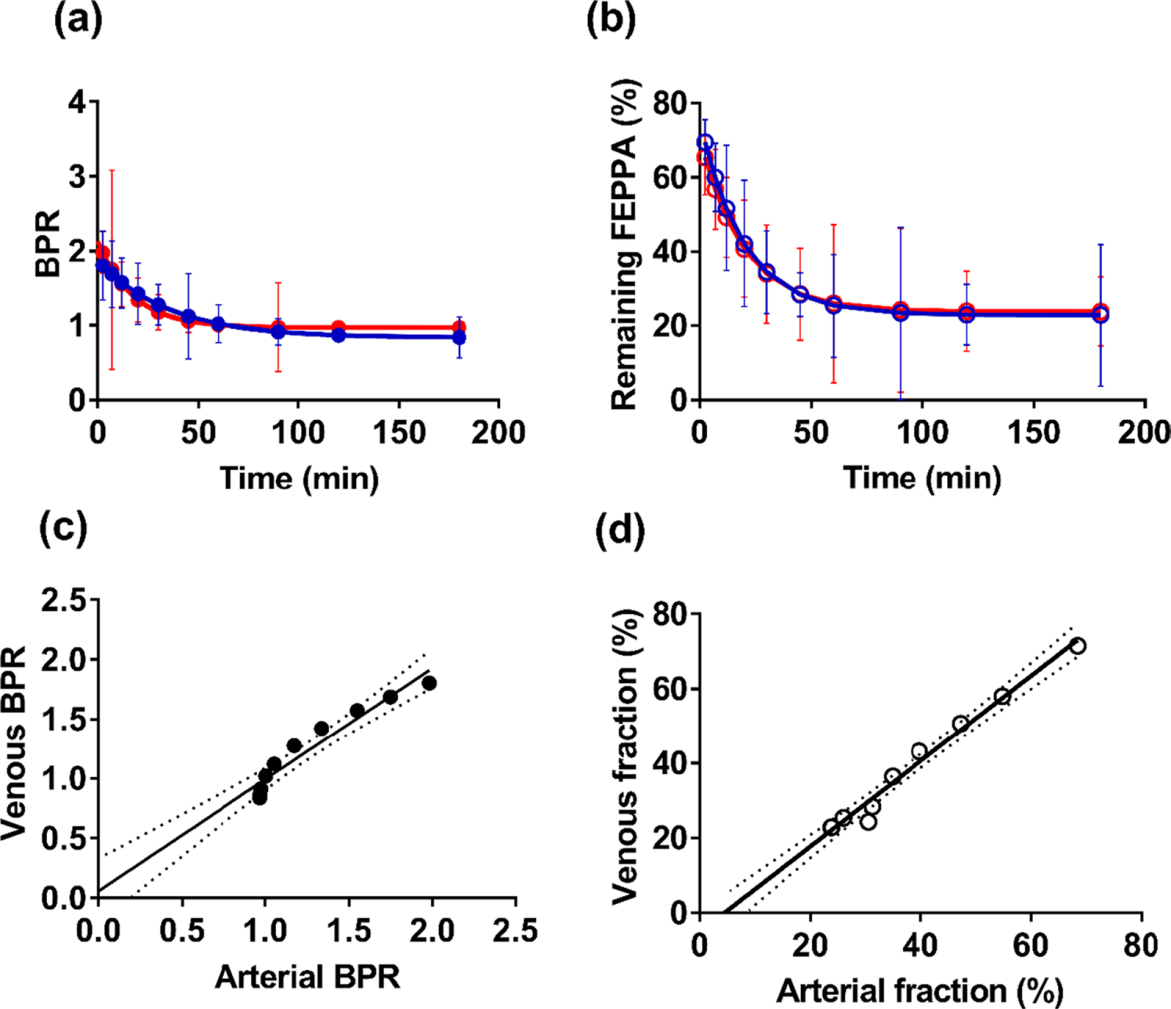
**a** Blood-to-plasma ratios (BPRs) from arterial (red filled) and venous (blue filled) blood samples (results are from 8 animals). **b** Corresponding [^18^F]FEPPA plasma percent determined from venous (blue hollow) and arterial (red hollow) samples. Each value represents the mean ± standard deviation and are presented for 10 time points ranging from 2.5 to 180 min. **c** Linear correlation between venous and arterial BPRs (*R*^2^ = 0.93, slope = 0.94, intercept = 0.06). **d** Correlation between remaining [^18^F]FEPPA fraction from venous and arterial blood samples (*R*^2^ = 0.98, slope = 1.14, intercept = − 5.11). Note that figures **c** and **d** appear to show only nine points because of the overlap of two datasets (specifically at arterial BPR = 0.97, venous BPR = .85 and arterial fraction = 24, venous fraction = 23)

Figure 4 presents average BRPs and remaining [^18^F]FEPPA fractions derived separately from venous and arterial blood samples. Linear correlation analysis indicated very strong agreement between arterial and venous sampling for both BPR (*R*^2^ = 0.93, *p* < 0.001) and remaining [^18^F]FEPPA fraction (*R*^2^ = 0.98, *p* < 0.001). In both cases, the regression slope was not significantly different from a value of one.

### Feasibility study

**Fig. 5:**
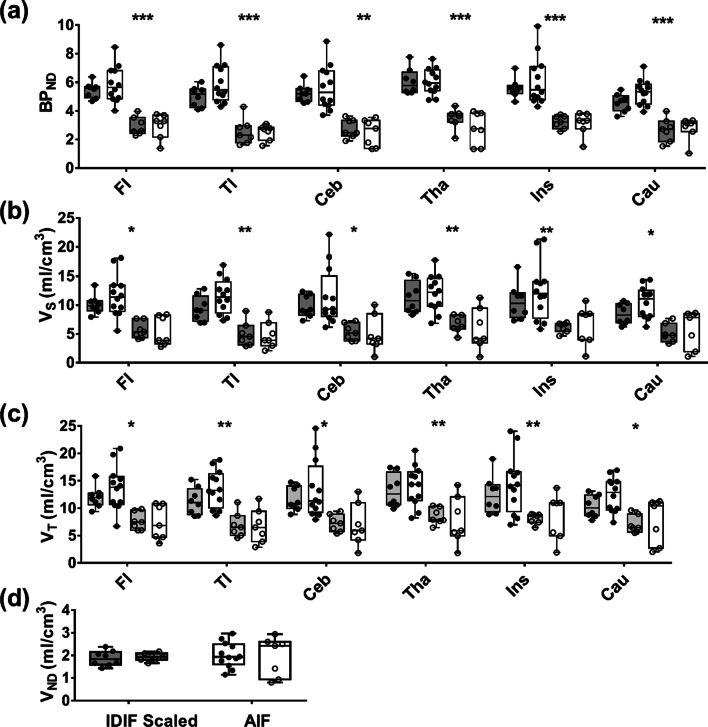
Results from applying SIME to data from healthy controls in which the AIF was measured directly (clear box plots) and those with venous metabolite-corrected IDIFs (gray box plots): **a** regional BP_ND_, **b** regional *V*_S_, **c** regional *V*_T_ and **d** common *V*_ND_. Data are presented as box–whisker plots with the median indicated by a black line. In all cases, data were divided into HABs (filled circles) and MABs (open circles). Significant between HABs and MABs are indicated by asterisk (*p < 0.05, **p < 0.01 and ***p < 0.001)

No significant differences in regional BP_ND_ estimates were found between the two groups of healthy participants, i.e., those used to determine the accuracy of SIME in which the AIF was directly measured and those from the minimally invasive group that involved venous metabolite-corrected IDIFs (Fig. 5a). In agreement with the accuracy of SIME section, BP_ND_ estimates derived using IDIFs were significantly greater for HABs compared to MABs (44 ± 7%). Similarly, regional *V*_S_ estimates (Fig. 5b) were significantly greater for HABs compared to MABs for both IDIFs (43 ± 5%) and AIFs (51 ± 10%) and regional *V*_T_ estimates (Fig. 5c) were significantly greater for HABs compared to MABs for both IDIFs (36 ± 3%) and AIFs (44 ± 5%); however, no significant differences were found between regional measurements from the two groups. Finally, there was no statistical difference between mean *V*_ND_ estimates (Fig. 5d) from the two groups: *V*_ND_ = 1.90 ± 0.33 mL/cm^3^ for the IDIF group and 2.02 ± 0.55 mL/cm^3^ for the AIF group.

## Discussion

Noninvasive quantification of TSPO uptake in the brain by PET is challenging due to the lack of a suitable anatomical reference region. The necessity for arterial blood sampling to measure the AIF is particularly concerning when working with clinical populations with cognitive and behavioral challenges, such as patients with dementia and other neuropsychiatric disorders. This study investigated a minimally invasive SIME-based approach aimed at simplifying the steps required to quantify regional [^18^F]FEPPA uptake. The main findings were that SIME enabled the imaging duration to be reduced to 90 min from the original 2–3 h required for full kinetic analysis [2] and accurate estimates of regional BP_ND_ and *V*_S_ were generated by SIME using IDIFs that incorporated venous metabolite correction.

To evaluate the accuracy of SIME, it was applied to a [^18^F]FEPPA dataset that included metabolite-corrected AIFs in order to compare *V*_ND_ and *V*_T_ estimates to those obtained by standard kinetic modeling. All regional *V*_ND_ values were within ± 22% of the overall average. Furthermore, there was no significant difference between *V*_ND_ estimates from SIME and the 2TCM, suggesting that the assumption of a uniform *V*_ND_ across brain regions is reasonable for [^18^F]FEPPA. The accuracy of estimating *V*_ND_ will depend on selecting an appropriate number of ROIs with a range of kinetic behaviors [6, 19]. For this study, ROIs were selected based on literature values of the rate constants for [^18^F]FEPPA [2] and visible differences in their TACs. Permutation analysis demonstrated that *V*_ND_ estimates with similar accuracy could be obtained for four, five, and six ROIs; however, significantly better precision was achieved with six.

Similar to the *V*_ND_ results, good agreement was found between SIME and the 2TCM with respect to regional *V*_T_. No significant differences between estimates from the two approaches were found in any of the six ROIs analyzed, and both approaches detected significantly larger *V*_T_ values for HABs compared to MABs. These results are in good agreement with Schain et al., 2018 who used SIME for quantification with the second-generation TSPO tracer [^11^C]PBR28 [7]. A further advantage of SIME is that by reducing the number of fitting parameters, the inter-subject variability was reduced by roughly 40% compared to the 2TCM. This result agrees with previous studies involving multiple tracers that reported greater reliability in parameter estimation with SIME [6, 7, 20].

By taking advantage of the greater precision provided by SIME, the possibility of reducing the scan duration required for accurate [^18^F]FEPPA quantification was investigated. The results presented in Fig. 2 demonstrate that reducing the scan time from 120 to 90 min had no significant effect on the accuracy of either regional BP_ND_ or brain-wide *V*_ND_. Furthermore, BP_ND_ exhibited the same dependency on genotype as *V*_T_. The ability to estimate regional BP_ND_ precisely was confirmed by the Monte Carlo simulations (Fig. 3). These simulations indicated that for average residuals based on measured TACs, only small differences were found between the average estimates of BP_ND_ and the corresponding input values (average bias of 1 ± 0.3%). A potential limitation of this error analysis is that Gaussian noise does not account for possible covariance between TACs from separate ROIs. To assess potential covariance, the correlation between residuals from different TACs was calculated. The average correlation was weak to moderate (0.33 ± 0.42), indicating that the noise model was reasonable. An alternative approach would be to use empirical residuals in the simulations as proposed by Plavén-Sigray et al.; however, the number of datasets in the current study was not large enough to generate a sufficient number of simulations [8].

The application of SIME, such as with [^11^C]PBR28, has typically involved using a population-based AIF [6, 7]. A disadvantage of this approach is the possibility of changes to the shape of the AIF related to binding affinity or disease [9, 18]. In the current study, an alternative minimally invasive approach was investigated that involved extracting the IDIF for each participant and using serial venous blood samples for metabolite correction. Mabrouk et al. [21] demonstrated that accurate estimates of regional *V*_T_ could be obtained using IDIFs scaled by an arterial blood sample. Based on these results, the current study used IDIFs scaled to late venous samples (i.e., 45, 60, and 90 min) when arterial and venous [^18^F]FEPPA concentrations are expected to be in equilibrium. Serial venous samples were also used for metabolite correction, as confirmed in the animal study that showed good agreement between arterial and venous measures of BPR and remaining [^18^F]FEPPA fraction (Fig. 4). The greatest error was found at the earliest sampling time, indicating that venous and arterial [^18^F]FEPPA concentrations had yet to reach equilibrium [20]. However, Fig. 4 illustrates that the magnitude of the error was relatively small (i.e., 10.8 ± 6.2% for the remaining [^18^F]FEPPA fraction at 2.5 min). This was confirmed by comparing BP_ND_ estimates from the two groups of healthy participants. No significant difference in BP_ND_ was found in any of the ROIs and there was good agreement between the two groups in terms of the difference between HABs and MABs. The need for venous sampling does add complexity to the imaging procedure. A possible solution would be to incorporate a model of the metabolite pool into SIME, although this comes at the cost of increasing the dimensionality of the cost function [22]. Note that these animal studies did not account for possible variations in the shape of the AIF due to TSPO polymorphisms; however, there is no evidence of polymorphisms in pigs [23].

Although BP_ND_ has the advantage of being insensitive to scaling errors in the input function, recent studies have reported greater variability in BP_ND_ compared to *V*_S_ [8]. Furthermore, *V*_ND_ has been found to change in some patient populations, which would be a confounder for interpreting changes in *V*_T_ and suggests that *V*_S_ is a more appropriate marker of TSPO binding [9]. By collecting venous blood samples in the current study, it was possible to scale individual IDIFs in order to estimate regional *V*_S_. Similar to BP_ND_, *V*_S_ was found to be sensitive to genotype and regional estimates obtained by the minimally invasive approach were in good agreement with those obtained from the dataset that included AIFs.

A limitation of this work was that the feasibility study did not involve measuring the AIF and consequently, validating the minimally invasive SIME was based on a comparison to the retrospective dataset. The good agreement in terms of regional BP_ND_ and *V*_S_, as well as brain-wide *V*_ND_ from the two datasets, indicates that the minimally invasive approach can measure these parameters accurately. Another consideration is the age difference between participants in the two datasets; the retrospective data consisted of participants in middle age, while the mean age of the participants recruited for the minimally invasive imaging protocol was above 70 y. Despite this age difference, normal aging is not associated with increased [^18^F]FEPPA uptake [24]. A final limitation was the lack of a patient population to investigate if the approach is sensitive to disease-specific changes in TSPO binding. The consistently greater values of *V*_T_, *V*_S_, and BP_ND_ in HABs compared to MABs demonstrate that the approach is sensitive to changes in specific binding; however, it would be prudent to investigate its accuracy in patient populations.

## Conclusions

This study demonstrated that quantifying regional [^18^F]FEPPA uptake could be performed without arterial blood sampling and with a shorter scan time by using SIME. Collecting serial venous blood samples enabled accurate correction of [^18^F]FEPPA metabolites and provided a means of scaling the IDIF for kinetic modeling. Using SIME to estimate *V*_ND_ across brain regions enabled accurate and precise estimates of regional specific binding (i.e., BP_ND_ and *V*_S_) from 90-min TACs compared to the 2–3 h required for the unconstrained 2TCM.

## Data Availability

Access to the data and scripts is available from the corresponding author upon reasonable request.
